# Reoccurring *Salmonella* Cotham Outbreak Linked to Pet Bearded Dragons — United States, 2024

**DOI:** 10.15585/mmwr.mm7431a1

**Published:** 2025-08-21

**Authors:** Lingzi Xiaoli, Paula Huth, Cassandra Sherman, Danielle Wroblewski, Stacy Anderson, Lindsey Ferraro, Dee Jones, Molly Slaughter, Brenda Morningstar, Tonya Mackie, Natalee Bowen, Molly Leeper, Katie Werner, Katharine Benedict, Kate Varela

**Affiliations:** ^1^Division of Foodborne, Waterborne, and Environmental Diseases, National Center for Emerging and Zoonotic Infectious Diseases, CDC; ^2^New York State Department of Health; ^3^New York State Department of Health, Buffalo, New York; ^4^Southeastern District Health Department, Dothan, Alabama; ^5^Alabama Department of Public Health; ^6^Minnesota Department of Health; ^7^National Veterinary Services Laboratories, Animal and Plant Health Inspection Service, U.S. Department of Agriculture, Ames, Iowa; ^8^ASRT Inc., Atlanta, Georgia.

**Summary**

**What is already known about this topic?**

Bearded dragons have been linked to *Salmonella* outbreaks, including a 2012–2014 *Salmonella* Cotham outbreak involving 160 patients in 35 states.

**What is added by this report?**

Twenty-seven cases (26 confirmed and one probable) of *S*. Cotham infection were identified during January 11–December 10, 2024. Eighteen reported cases were among persons with household exposure to bearded dragons; *S.* Cotham isolates from two bearded dragons kept in two patient households were closely genetically related to each other, to 2012–2014 isolates, and to clinical isolates from the 26 confirmed cases. Children aged <5 years, especially infants, were disproportionately affected.

**What are the implications for public health practice?**

Bearded dragons pose a continued risk for *Salmonella* infection. Owners might reduce the risk by limiting reptiles’ free roaming and by washing hands and changing clothing after handling the reptile and before interacting with an infant or young child.

## Abstract

In April 2024, CDC’s PulseNet identified a cluster of seven *Salmonella* Cotham cases from five states. Isolates were highly related by whole genome sequencing (WGS), and one patient reported contact with a pet bearded dragon. CDC initiated a multistate investigation and as of December 10, 2024, an additional 19 cases had been identified, for a total of 26 confirmed cases from 13 states; state public health partners identified one probable case in an additional state for a total of 27 cases. Eighteen of 25 cases (72%) were among persons who reported contact with a bearded dragon or lizard. Children aged <5 years, especially infants, were disproportionately affected, accounting for 17 (65%) of the 26 confirmed cases; most had bearded dragons in the home without direct animal contact. WGS of two bearded dragon specimens collected in 2024 and three bearded dragon specimens collected during 2012–2014 confirmed genetic relatedness of this rare *Salmonella* strain and continued circulation among commercially sold bearded dragons. CDC implemented a One Health approach in response, working with pet industry representatives to disseminate information about biosecurity best practices to bearded dragon suppliers and retailers. Investigators contacted a common bearded dragon supplier identified in the traceback investigation to share biosecurity and prevention recommendations. CDC used social media and a website investigation notice to inform the public, recommending that caregivers prevent young children from indirect reptile contact by restricting reptiles from roaming freely, separating reptiles and supplies from food preparation areas, and washing hands and changing clothes after handling reptiles and before holding infants.

## Investigation and Outcomes

### Identification of First Seven Cases

In April 2024, CDC’s PulseNet* (*1*), the national molecular subtyping network for enteric disease surveillance, identified a cluster of seven *S.* Cotham illnesses from five states; isolates from these cases were found by WGS to be highly related. *S.* Cotham, a rare *Salmonella* serotype, was linked to bearded dragons during a 2012–2014 outbreak involving 160 patients from 35 states (*2*). In the 2024 cluster of seven patients, one patient reported contact with a pet bearded dragon, indicating a potentially reoccurring *S*. Cotham strain (i.e., a strain causing acute outbreaks separated by periods when illnesses are not detected).^†^ CDC initiated a multistate investigation to identify additional cases and exposures (*3*).

### WGS Identification of Additional Cases and Animal Investigations

PulseNet data analysts monitored the PulseNet database for additional *S.* Cotham illnesses related by WGS. State and local health departments conducted initial routine interviews as patients with *S.* Cotham infection were identified. Those who reported recent reptile contact were reinterviewed by administration of a standardized supplemental questionnaire that collected additional information, including detailed reptile exposure information. The supplemental questionnaire included questions regarding reptile species, behavioral practices, ownership, and purchase location, which investigators used to gather bearded dragon retailer and supplier traceback information. Initial routine interviews and interviews with the supplemental questionnaire were attempted for the seven patients initially identified by PulseNet and any additional patients identified throughout the investigation. State and local health departments conducted animal and environmental sampling at patient residences for those who reported owning a bearded dragon and were willing to have their pet tested for *Salmonella*. In a collaborative effort, the U.S. Department of Agriculture’s National Veterinary Services Laboratory performed WGS in 2024 of bearded dragon isolates collected during the 2012–2014 outbreak. This activity was reviewed by CDC, deemed not research, and was conducted consistent with applicable federal law and CDC policy.^§^

As of December 10, 2024, when the investigation was closed, PulseNet had identified an additional 19 cases, for a total of 26 confirmed cases in 13 states (Table 1) using WGS, and state public health partners identified one probable^¶^ case in an additional state for a total of 27 cases in 14 states.

**TABLE 1:** Demographic characteristics of patients* with *Salmonella* Cotham illnesses linked to bearded dragons, among patients aged ≤1–67 years — United States, 2024

Characteristic	No. (%)
**Sex (n = 26)**	
Female	17 (65)
Male	9 (35)
**Race (n = 25)**	
Black or African American	3 (12)
White	17 (68)
More than one race reported	2 (8)
No race reported	2 (8)
Unknown	1 (4)
**Ethnicity (n = 26)**	
Hispanic or Latino	7 (27)
Non-Hispanic	18 (69)
Unknown	1 (4)
**State of residence (n = 27)**	
Alabama	1 (4)
California	2 (7)
Colorado	1 (4)
Georgia	3 (11)
Iowa	1 (4)
Minnesota	1 (4)
New York	4 (15)
North Carolina	2 (7)
Ohio	4 (15)
Oklahoma	2 (7)
Pennsylvania	2 (7)
Tennessee	1 (4)
Texas	2 (7)
Washington	1 (4)
**Hospitalization (n = 24)**	
Yes	10 (42)
No	14 (58)

* Patients had confirmed (26) or probable (one) infection. A confirmed case was defined as a fecal, urine, or blood *Salmonella* Cotham isolate related to outbreak cluster isolates with isolation dates on or after January 11, 2024. A probable case was defined as the occurrence of clinical signs consistent with salmonellosis on or after January 11, 2024, in a person with exposure to a bearded dragon, and a polymerase chain reaction–confirmed *Salmonella* positive test result with no isolate available for whole genome sequencing. Age, sex, race, ethnicity, and hospitalization data were not available for the probable case. Race information was missing for one confirmed case, and hospitalization status was unknown for two confirmed cases.

### Characteristics of Patients with Confirmed and Probable Cases

A confirmed case was defined as *S*. Cotham infection with a fecal, urine, or blood isolate related to outbreak cluster isolates, and isolation dates on or after January 11, 2024. Observed allele differences among isolates ranged from 0 to 13 and were based on core genome multilocus sequence typing (cgMLST), a WGS-based analysis method used to compare the genomes of bacterial strains (*4*). Confirmed case illness onset dates ranged from January 8 to October 31, 2024. Median patient age among 26 of the 27 cases with known ages was 1 year (range = ≤1–67 years). Seventeen of 26 patients (65%) with confirmed illness were children aged <5 years; 13 (50%) were infants aged <1 year. Ten of 24 patients (42%) with confirmed illness were hospitalized; hospitalization status was unknown for two patients with confirmed illness. Thirteen of 14 patients with confirmed illness who completed supplemental questionnaires reported gastrointestinal symptoms including diarrhea.

A probable case of *S*. Cotham infection was defined as the occurrence of clinical signs consistent with salmonellosis on or after January 11, 2024, in a person with exposure to a bearded dragon, and polymerase chain reaction–confirmed *Salmonella* positive test result but with no isolate available for WGS; however, in the one identified probable case, feces of the bearded dragon owned by the patient was tested and resulted in isolation of the *S*. Cotham outbreak strain. Detailed demographic and illness outcome information were not available for the patient with probable infection.

### Reptile Exposures

Detailed reptile exposure information was ascertained from the initial public health interview and supplemental questionnaire. In the initial interview, 18 of 25 patients (72%) reported a reptile in the household where they lived or visited within 7 days before illness onset; 11 of the 18 patients were aged <5 years. Reported reptile species included 17 bearded dragons and one lizard without species information that was associated with a patient aged <1 year. Of the 18 patients who reported a reptile in the household, nine proxies (i.e., parents or caregivers of patients aged <5 years) from separate families reported that their children did not have direct contact with the bearded dragon or lizard (i.e., did not pet, touch, or hold the reptile; did not feed the reptile by hand; and were not licked, scratched, or bitten by the reptile) but had contact with caregivers or household members who touched or held the bearded dragon or touched its enclosure (Table 2). Parent responses also indicated bearded dragon contact among children aged <5 years occurred via indirect animal contact, defined as exposure to contaminated areas where animals lived and roamed, contaminated objects or surfaces, or contact with a person (e.g., a parent or caregiver) who had contaminated hands or clothing, but not the animal itself.

**TABLE 2:** Bearded dragon reported in household, by age group and reported contact type, during *Salmonella* Cotham investigation — United States, 2024

Age group, yrs	No. (%)	Bearded dragon in household			Contact type among persons with bearded dragon in household*		
		No	Unknown	Yes	Direct^†^	Indirect^§^	Unknown^¶^
<1	13 (50)	6	—	7**	—	6	1
1–4	4 (15)	—	—	4	—	3	1
5–18	4 (15)	—	—	4	1	3	—
19–39	2 (8)	—	1	1	1	—	—
40–64	2 (8)	1	—	1	1	—	—
65–67	1(4)	—	—	1	1	—	—
**Total**	**26 (100)**	**7**	**1**	**18**	**4**	**12**	**2**

* Contact type for the 18 instances of known reptile exposure refers to how the reptile exposure occurred: direct, indirect, or unknown type, ascertained by information shared by the patient or proxy (i.e., parent or caregiver) during the initial public health interview or responses to the supplemental reptile exposure questionnaire.

^†^ Direct contact refers to exposure to the saliva, blood, urine, mucous, feces, or other body fluids of an infected animal that occurs when people pet, touch, or hold an animal, feed an animal by hand, or are licked, scratched, or bitten by an animal.

^§^ Indirect contact is defined as exposure to areas where animals live and roam, or contaminated objects or surfaces, and not the animal itself. Indirect contact might also include contact with another person (e.g., a parent or caregiver) who has contaminated hands or clothing.

^¶^ The parents of two patients aged <1 year reported having a bearded dragon in the household during the initial interview but were lost to follow-up for reinterview with the supplemental reptile exposure questionnaire, and no additional information was available to determine if the patients had direct or indirect contact.

** The parent of one patient aged <1 year reported their infant was in a household with a lizard of unknown species.

Ten patients or their proxies who reported reptile exposure were reinterviewed with the supplemental questionnaire to obtain information on reptile ownership and practices, including one parent representing each of six patients aged <5 years from different households. Two of the six parents reported that the bearded dragon in each respective household was allowed to roam freely, with one specifying the reptile was allowed in bed. Four parents provided information on hand hygiene behaviors, with two parents reporting always washing their hands after handling bearded dragons and two reporting almost always. Two parents reported cleaning baby bottles and bearded dragon supplies in the same sink. All six parents of patients aged <5 years who were reinterviewed with the supplemental questionnaire reported they did not have prior knowledge of reptile-associated salmonellosis and were unaware that reptiles can transmit *Salmonella*.

### Bearded Dragon Sampling Results and Supplier Traceback 

Analysis of fecal, rectal, and environmental specimens collected from two bearded dragons and their habitat in the 2024 outbreak, representing two different patient households in Alabama and Minnesota, resulted in three *S.* Cotham isolates that matched the outbreak strain. WGS using PulseNet’s predefined cgMLST allele scheme (*4*) demonstrated that five *S.* Cotham isolates obtained from individual bearded dragons during the 2012–2014 (three) and 2024 (two) investigations were closely genetically related (i.e., within 0–13 allele differences by cgMLST) to each other and to clinical isolates (26) collected during the 2024 investigation (Figure). Traceback information gathered through the supplemental questionnaire indicated that recently purchased bearded dragons within the households of four 2024 patients shared a common bearded dragon supplier. This 2024 common supplier was not identified during the 2012–2014 investigation, and information was not available to determine whether this common supplier sourced bearded dragons or breeding stock from breeders that were identified during 2012–2014.

**FIGURE:**
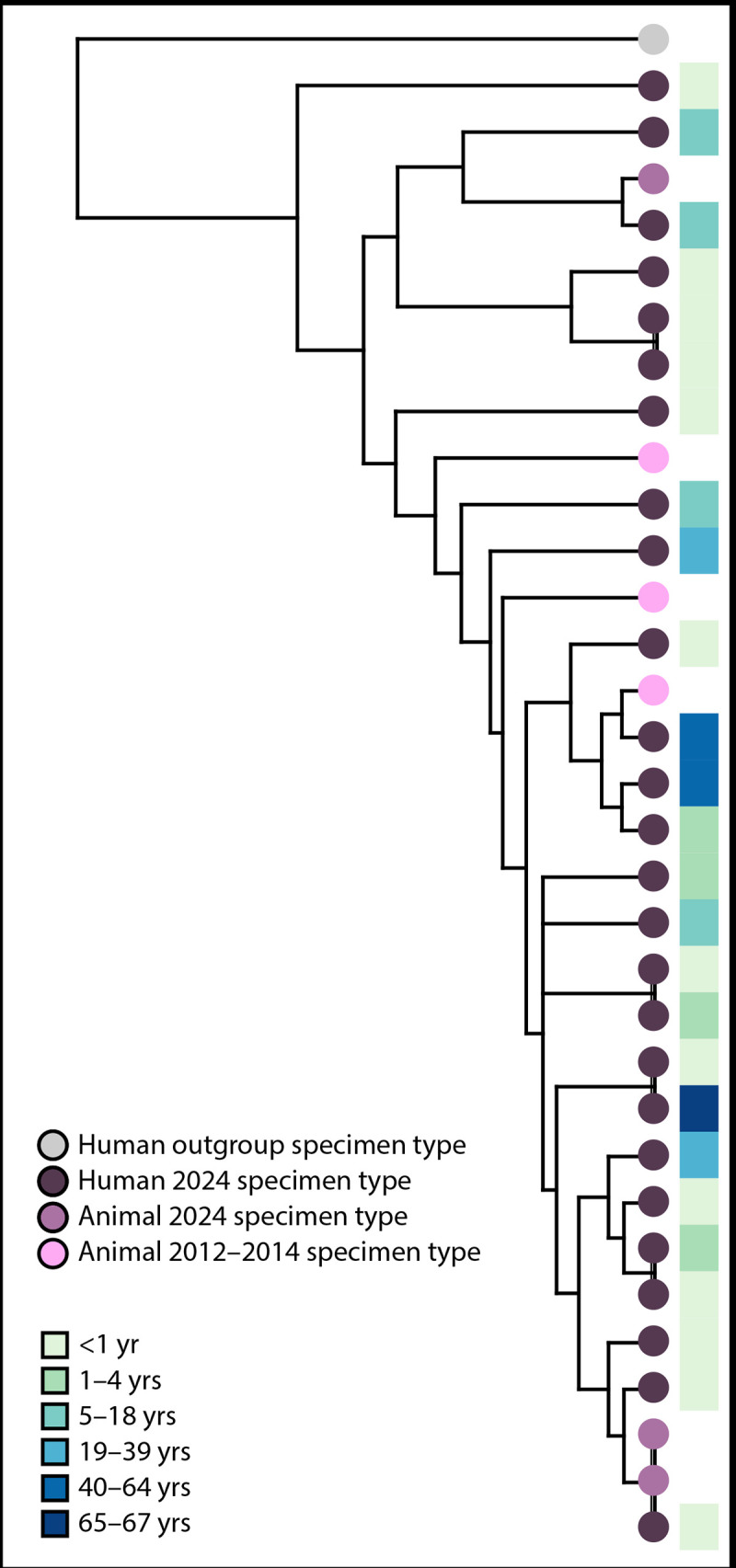
Phylogenetic tree* of 32 genetically closely related *Salmonella* Cotham isolates belonging to two outbreaks linked to bearded dragons, by age group and specimen type — United States, 2012–2014 and 2024 **Abbreviation:** cgMLST = core genome multilocus sequence typing. * This phylogenetic tree was constructed using the PulseNet 2.0 cgMLST allele scheme and annotated in iTOL (version 5). All outbreak-associated isolates are related to each other within 0–13 alleles by cgMLST, with the outgroup isolate differing by up to 42 alleles. The tree demonstrates overlap in genetic relatedness between the 2012–2014 outbreak and the 2024 outbreak. One *Salmonella* Cotham outgroup, distinct from the outbreak cluster based on its larger cgMLST allele differences, is included to root the tree and illustrate evolutionary differences in the outbreak isolates.

## Discussion

This report describes an outbreak of illnesses of *S.* Cotham linked to bearded dragons 10 years after *S.* Cotham was first found to be associated with gastrointestinal illness from bearded dragon exposure (*2*). Epidemiologic, laboratory, and traceback evidence all support household exposure to pet bearded dragons as the *S.* Cotham source. During the 10 years since the 2012–2014 outbreak, the genetic diversity of *S.* Cotham might have been expected to expand; however, no other *S.* Cotham clusters were detected. Given that *S.* Cotham is a rare *Salmonella* serotype and known to be previously linked to reptile exposure, the close genetic relatedness among isolates collected during 2012–2014 and 2024 indicate a potential reoccurrence of the strain and common vehicle across both investigations. The reasons for reoccurrence of this strain in bearded dragons is unclear, underscoring the need for a better understanding of pet supply chains and the ecology of *Salmonella* in these environments.

Infants aged <1 year were disproportionately affected in this outbreak (50%) compared with a 10-year summary of multistate reptile- and amphibian-associated non-Cotham salmonellosis outbreaks in which 19% of patients were aged <1 year (*5*). Parent responses to reptile exposure questions indicated that most exposures among children aged <5 years occurred via indirect animal contact (Table 2). Caregivers can prevent young children from indirect reptile contact by restricting reptiles from roaming freely; separating reptiles and supplies from food preparation areas; and after handling reptiles, washing hands and changing clothes before holding infants and young children.

A multicomponent One Health** approach in response to this outbreak was implemented. Investigators alerted pet industry representatives to disseminate information about the outbreak and biosecurity best practices to their national network of bearded dragon suppliers and retailers. Investigators also contacted the common supplier to share biosecurity and prevention recommendations including best practices for record keeping, cleaning and disinfection, employee education, and providing educational materials for customers (*6*). Pediatricians are encouraged to include a question on patient information forms that asks if reptiles are kept in the household to flag a conversation about *Salmonella* prevention (*6*) and to share educational handouts (e.g., Pet Patient Handout) with parents and caregivers of young children. Veterinarians are also encouraged to educate reptile owners about zoonotic disease risks and prevention measures. CDC used social media and a website investigation notice to inform the public, including an Instagram post that received 151,133 impressions and leveraged engagement from a popular television series, to uniquely reach a different audience than typical posts. Parents and caregivers can prevent young children from indirect reptile contact by restricting reptiles from roaming freely; separating reptiles and supplies from food preparation areas; and after handling reptiles, washing hands and changing clothes before holding infants and young children. Physicians, veterinarians, and pet industry professionals are encouraged to share infection risk information and prevention recommendations to help owners safely enjoy their pets without becoming ill (*3*,*6*).

## Footnotes

*
Outbreak Detection | PulseNet | CDC

†
Reoccurring, Emerging, and Persisting Enteric Bacterial Strains | Foodborne outbreaks | CDC

§ 45 C.F.R. part 46, 21 C.F.R. part 56; 42 U.S.C. Sect. 241(d); 5 U.S.C. Sect. 552a; 44 U.S.C. Sect. 3501 et seq.

¶ A probable case of *S*. Cotham infection is the occurrence of clinical signs consistent with salmonellosis on or after January 11, 2024, in a person with exposure to a bearded dragon, and polymerase chain reaction–confirmed *Salmonella* positive test result with no isolate available for WGS. In the probable instance, feces of the bearded dragon owned by this patient were tested and resulted in isolation of the *S*. Cotham outbreak strain.

**
One Health | CDC
